# Associations between identity perception, symptom severity, and quality of life in adolescents with ADHD

**DOI:** 10.1038/s41598-025-26963-5

**Published:** 2025-11-28

**Authors:** Adi Stern, Liron Lamash

**Affiliations:** 1 Department of Occupational Therapy, Ben-Gurion University of the Negev, Beer-Sheva, 8410501 Israel; 2 Department of Occupational Therapy, University of Haifa, Haifa, 3498838 Israel

**Keywords:** ADHD, Adolescence, Illness identity, Quality of life, Diseases, Health care, Medical research, Psychology, Psychology

## Abstract

Adolescence is a key stage for identity formation. Attention deficit hyperactivity disorder (ADHD) is a lifelong neurodevelopmental condition that significantly affects adolescents’ daily functioning and quality of life and plays an important role in shaping their identity. Illness identity refers to the extent to which a chronic diagnosis is integrated into an individual’s self-concept. This study examined how ADHD symptom severity relates to diagnosis identity dimensions and health-related quality of life (HRQoL) in adolescents with ADHD. A cross-sectional study was conducted with 154 adolescents aged 11 to 18 years (*M* = 15.40, *SD* = 1.83). Participants completed validated measures of ADHD symptom severity, diagnosis identity (*engulfment*, *rejection*, *acceptance*, *enrichment*), and HRQoL. Statistical analyses included correlations, regression, and mediation models to clarify the relationships among these variables. Acceptance was significantly higher than other identity dimensions, while engulfment and rejection were lower. Higher ADHD symptom severity, especially inattention, was linked to greater engulfment. Engulfment predicted lower quality of life more strongly than symptom severity itself, and positive social experiences supported healthier identity integration. Mediation analysis showed that only engulfment significantly explained the link between symptom severity and HRQoL. Findings highlight the role of diagnosis identity, particularly engulfment, in the well-being of adolescents with ADHD. Interventions that combine symptom management with identity-focused approaches and social support may more effectively enhance adolescents’ outcomes.

## Introduction

Attention deficit hyperactivity disorder (ADHD) is a common neurodevelopmental condition characterized by inattention and/or hyperactivity-impulsivity that interferes with functioning and development^1^. It is typically diagnosed in childhood and continues into adolescence and adulthood^2,3^. The global prevalence of ADHD among children and adolescents is estimated to be around 8%^4^. However, other studies have reported different rates by age group, with a prevalence of approximately 7.6% in children under 12 years old and 5.6% in adolescents 12 to 18 years old^5^.

Research has indicated that ADHD symptoms typically decline with age^6,7^. Clinical and epidemiological studies have shown that hyperactive-impulsive symptoms are more prominent in childhood and tend to decrease during adolescence, whereas inattentive symptoms tend to persist^8^. However, this decline often reflects not the resolution of symptoms but a developmental shift in their manifestation. Specifically, observable motor restlessness, such as fidgeting, running, or climbing excessively, tends to diminish during adolescence, whereas adolescents increasingly report experiencing internal restlessness, characterized by feelings of agitation and an inability to relax^9,10^. This pattern has been acknowledged in large-scale epidemiological surveys, suggesting that age-related changes in ADHD symptoms often involve a shift from overt behavioral symptoms to more internalized experiences^11,12^. In contrast, inattention symptoms persist and remain relatively stable during adolescence^8,13^.

Adolescence, typically defined as the period between the ages of 10 and 19 years, is a transitional phase between childhood and adulthood. It is marked by rapid and significant physical, cognitive, and psychosocial changes^14^. For adolescents with chronic health conditions or neurodevelopmental disorders, this developmental period often presents additional difficulties^15^. Typical ADHD symptoms, such as inattention, restlessness, and disorganization, further complicate this already challenging transition^16,17^. Although some visible symptoms may decline during adolescence, ADHD continues to have a substantial impact on daily functioning^18^. These difficulties often manifest in various domains, including academic underachievement, increased risk of school dropout, and social challenges such as peer rejection, difficulty forming relationships, and reduced participation in verbal interactions^19,20^.

Identity development is a central task of adolescence and young adulthood, as individuals shape their sense of self and envision their future societal roles^21,22^. Daily experiences and interactions influence this process, which is key in health, well-being, and social participation. Research has indicated that adolescents experiencing chronic health conditions or neurodevelopmental challenges may experience difficulties in identity formation, leading to feelings of alienation, personal suffering, and broader societal costs^23^.

Adolescents with ADHD may be particularly vulnerable to challenges in identity development. Studies have suggested that living with an ADHD diagnosis impacts how adolescents construct their personal narratives and understand themselves^23–26^. For some, ADHD becomes a central aspect of their identity, shaping their sense of uniqueness but also exposing them to vulnerability^27^. Others view ADHD as an external medical condition causing difficulties, but not integral to who they are. In contrast, some perceive the diagnosis merely as a label imposed by others^24^. Despite these insights, current knowledge about how adolescents with ADHD perceive their diagnosis remains limited. Most existing studies are qualitative and based on small samples^23,25,26,28^. A need for broader quantitative research exists to deepen our understanding of ADHD-related identity perception in this population. Such understanding is crucial because how adolescents relate to their diagnosis can influence how they manage their condition, perceive future opportunities, and engage with the world^23,29^.

The concept of *illness identity* - or *diagnosis identity* when referring to ADHD - describes how a chronic health condition becomes part of an individual’s sense of self, influencing daily life, social relationships, and self-esteem. This integration shapes how individuals cope with everyday challenges and navigate life transitions^15,30,31^. Based on this framework, Oris et al.^31^ developed the Illness Identity Questionnaire (IIQ) conceptualizes illness identity as a multidimensional construct encompassing four ways of relating to one’s condition: rejection, acceptance, engulfment, and enrichment. *Rejection* reflects an effort to deny, minimize, or distance oneself from the illness. Individuals high on this dimension tend to avoid acknowledging their condition or discussing it with others, perceiving it as something external to the self. For example, the item *“I refuse to see my illness as part of myself”*. *Acceptance* involves acknowledging the illness as a genuine but manageable aspect of life, without allowing it to dominate one’s sense of self. Individuals who show greater acceptance can recognize the limitations imposed by their condition while maintaining a balanced identity. For example, *“I accept being a person with an illness”*. *Engulfment* represents an identity that is overtaken by the illness, where the condition becomes central to one’s self-definition and daily experience. People high in engulfment often describe their illness as influencing most of their thoughts, feelings, and behaviors. For instance, *“My illness completely consumes me”*. *Enrichment* reflects the perception that living with the illness contributes to personal growth, insight, and strength. It encompasses viewing the condition as an experience that broadens one’s self-understanding and life perspective. For example, *“Because of my illness*,* I have become a stronger person”*.

The IIQ is designed to allow the substitution of the word “illness” with any specific diagnosis. It has been applied in research on adolescents and young adults with various chronic health conditions, including diabetes^32^, epilepsy^16^, celiac disease^33^, autism^34,35^, and schizophrenia^35^. Across these studies, higher levels of *acceptance* and *enrichment*, the positive dimensions of illness identity, have been consistently associated with favorable outcomes, such as greater social participation and improved health-related quality of life (HRQoL), whereas *rejection* and *engulfment* were associated with greater distress and poorer QoL.

An individual’s HRQoL refers to their subjective perception and evaluation of the most meaningful aspects of their life situation, including health status, daily functioning, social integration, and participation in age-appropriate activities. As a multidimensional construct, HRQoL captures not only physical health but also psychological, social, and functional well-being, making it a comprehensive measure of the impact of a health condition^9,36,37^. In adolescents with ADHD, symptom severity has been consistently associated with lower HRQoL, highlighting the disorder’s broad impact on life domains beyond clinical symptoms^9,38^. Despite this, research on how adolescents’ identity perception relates to HRQoL in the context of ADHD remains scarce.

In summary, ADHD is a lifelong neurodevelopmental condition that significantly affects adolescents’ daily functioning and plays an important role in shaping their identity and life trajectory. Despite the growing recognition of ADHD’s impact on personal development, limited research has examined how adolescents perceive their ADHD diagnosis and how this perception relates to their well-being. The IIQ provides a novel quantitative framework for examining how adolescents incorporate their diagnosis into their self-concept and how this process may be related to symptom severity and HRQoL. Gaining a better understanding of these associations may inform clinical practice and guide the development of tailored interventions to support adolescents in managing ADHD and its psychosocial implications. The current study had four main aims: (1) to describe the diagnostic identity profile of adolescents with ADHD; (2) to examine the associations between diagnostic identity perception and ADHD symptom severity and HRQoL; (3) to examine how ADHD identity and ADHD symptom severity predict overall QoL, and how ADHD symptom severity and HRQoL predict ADHD identity; (4) to test whether the diagnostic identity dimensions mediate the relationship between ADHD symptom severity and HRQoL.

## Methods

### Study design

This study employed a cross-sectional design using an anonymous online survey. Participants were recruited through convenience sampling via online advertisements in ADHD support groups and social media platforms.

### Participants

#### Power and sample size

A priori power analysis using G*Power software (version 3.1.9)^39^ indicated that 84 participants were required to detect a moderate correlation (*r* =.30, α = 0.05, power = 0.80). With 154 participants, the study had sufficient power for the regression models (f² ≈ 0.05) and, based on prior guidelines, adequate power to detect medium-sized indirect effects in the mediation analyses using bootstrapping.

#### Sample characteristics

The final sample included 154 adolescents aged 11 to 18 years (*M* = 15.40, *SD* = 1.83) with a formal diagnosis of ADHD. Of these, 101 participants (65.6%) identified as male, 52 (33.8%) as female, and 1 (0.6%) as nonbinary. Participants’ ADHD was diagnosed between the ages of 4 and 17 (*M* = 9.94, *SD* = 2.97) by a qualified health care professional (e.g., neurologist, psychiatrist, or pediatrician), as reported by the participants’ parents. At the time of the study, 80 participants (51.9%) reported current use of ADHD medication, whereas 74 participants (48.1%) were not receiving pharmacological treatment. Regarding educational placement, 120 adolescents (77.9%) were enrolled in regular mainstream classrooms, 8 (5.2%) attended specialized gifted programs in science or sports, and 26 (16.8%) studied in special education settings or vocational tracks with partial matriculation eligibility. Regarding residence, 123 participants (79.9%) lived in urban or suburban areas, and 31 (20.1%) resided in rural or peripheral regions. To ensure the study focused on the specific impact of ADHD on identity perception, adolescents with comorbid chronic health conditions (e.g., type 1 diabetes, epilepsy, inflammatory bowel disease), neurodevelopmental diagnoses (e.g., autism spectrum disorder, intellectual disability), and co-occurring mental health conditions (e.g., anxiety, depression) were excluded from the sample.

### Measures

#### Demographic questionnaire

A self-report demographic questionnaire was designed to support the study’s inclusion and exclusion criteria and to characterize the sample. The questionnaire collected information on participants’ age, gender identity, educational placement, and place of residence. Additionally, parents confirmed their child’s formal ADHD diagnosis and reported any additional chronic health conditions or neurodevelopmental diagnoses.

#### Illness identity questionnaire

The IIQ^31^ is a self-report measure that assesses how individuals integrate a chronic condition, such as ADHD, into their identity. The questionnaire includes 25 items, rated on a 5-point Likert scale ranging from 1 (*strongly disagree*) to 5 (*strongly agree*). The IIQ captures four theoretical dimensions of illness identity: *rejection*, *engulfment*, *acceptance*, and *enrichment*. Each dimension is scored by calculating the mean score of the relevant items. Higher scores indicate a more substantial presence of that identity component. A total illness identity score is computed by reverse-coding the negative dimensions (rejection and engulfment) and calculating the overall mean, whereby higher scores reflect a more adaptive and positive illness identity. The IIQ has demonstrated robust psychometric properties in clinical populations, including good internal consistency (α = 0.75–0.95) and factorial validity^15,31,35^. In the current study, the IIQ was adapted for ADHD by replacing the term “illness” with “ADHD diagnosis” following the authors’ guidelines^31^. Internal consistency in the present sample was acceptable (α = 0.72).

#### The ADHD Self-Report scale

The Adult ADHD Self-Report Scale (ASRS)^11^ is a widely used self-report instrument designed to assess current ADHD symptoms. It was developed by the World Health Organization^40^ and the Work Group on Adult ADHD^11^. The ASRS comprises 18 items, covering the core symptoms of inattention, hyperactivity, and impulsivity, as outlined in the *DSM-5* criteria^41^. Each item is rated on a 5-point Likert scale ranging from 0 (*never*) to 4 (*very often*). Clinically, the ASRS is used as a screening tool, with symptom counts based on the number of items rated 3 (*often*) or 4 (*very often*). A clinically significant symptom level is defined as endorsing six or more items at this threshold within either symptom domain (inattention or hyperactivity/impulsivity), corresponding to a minimum subscale score of 18^42,43^.

In research settings, particularly studies involving adolescents, the ASRS is frequently employed as a continuous measure of symptom severity. This approach involves summing the responses across all items to obtain a total symptom severity score (0–72) and calculating subscale scores for inattention (items 1–4, 7–11) and hyperactivity/impulsivity (items 5, 6, 12–18). Continuous scoring enables a more nuanced assessment of symptom variability and is a standard practice in adolescent sample studies^44–46^. In the current study, the ASRS was administered as a continuous measure, focusing on total and subscale severity scores rather than categorical symptom counts. The ASRS has demonstrated strong psychometric properties in adolescent populations, including good internal consistency (α = 0.79–0.89)^47^ and high discriminant validity between individuals with and without an ADHD diagnosis. The present sample’s internal consistency was satisfactory (α = 0.87).

#### Pediatric quality of life inventory

The Pediatric Quality of Life Inventory (PedsQL)^48^ is a validated self-report measure designed to assess HRQoL in children and adolescents. The questionnaire comprises 23 items that cover four domains of functioning: physical health, emotional well-being, social relationships, and school functioning^49^. Respondents rate the frequency with which they experienced difficulties in each area during the past month, using a five-point Likert scale ranging from 0 (*never a problem*) to 4 (*almost always a problem*). Following standard scoring procedures, items are reverse-coded and transformed to a 0 to 100 scale, whereby higher scores indicate better perceived quality of life (QoL; 0 = 100, 1 = 75, 2 = 50, 3 = 25, 4 = 0). Separate scores are computed for each domain, and a total HRQoL score represents overall functioning. The PedsQL has demonstrated robust psychometric properties in both clinical and community samples, including strong internal consistency and construct validity. It reliably differentiates between healthy individuals and those with acute or chronic health conditions^49^. In the present study, the internal consistency for the total adolescent report was good (α = 0.84).

### Procedure

The study was approved by the University of Haifa Ethics Committee (Approval No. 274/19 − 1) and conducted in accordance with the ethical standards of the institution. As the study employed anonymous, non-interventional convenience sampling and did not involve any personally identifiable or sensitive information, it met the criteria for exempt research under institutional regulations and Good Clinical Practice (GCP) guidelines. All procedures were performed in accordance with relevant institutional and international regulations, including the Declaration of Helsinki.

Participants were recruited via online outreach, including advertisements on social media platforms (e.g., Facebook and Instagram), relevant forums for parents of adolescents, and ADHD interest groups throughout Israel. Recruitment messages targeted parents and included detailed study information and a parental informed consent form. Parents who agreed to their child’s participation forwarded a secure link to the study on institutional secured Google Forms to their adolescent. Upon receiving the link, adolescents provided their assent by completing a digital assent form before participating in the study. A system of matching anonymous identification numbers was used to maintain confidentiality while linking parental consent to adolescent assent. All data were collected anonymously and stored securely to ensure participants’ privacy.

### Data analyses

Prior to conducting statistical analyses, the data were screened for quality. As all survey items were mandatory, no missing data occurred. All cases were manually reviewed to detect irregular or inconsistent response patterns, such as repetitive or uniform answers across multiple items, extremely short completion times, or contradictory responses across related items. No invalid or unreliable responses were identified; therefore, the full sample was retained for analysis. All analyses were performed using IBM SPSS Statistics (version 27). We first used descriptive statistics, including frequencies, ranges, means, and standard deviations, were calculated to characterize the sample and study variables. The internal consistency of each measure was assessed using Cronbach’s alpha coefficients. The four IIQ dimensions were analyzed as a within-subject repeated-measures factor analysis of variance (ANOVA) to allow comparison of adolescents’ relative endorsement of each dimension within the same individuals. Correlation analyses were conducted to examine associations between ADHD symptom severity, diagnostic identity, and HRQoL. Statistical significance was set at *p* <.05 for all analyses. Stepwise multiple regression analyses were performed to identify predictors of diagnostic identity and overall HRQoL. Finally, a parallel mediation analysis (PROCESS Model 4^50^) was conducted to test whether diagnostic identity dimensions mediated the association between ADHD symptom severity and HRQoL.

## Results

### ADHD identity perception

**Fig. 1:**
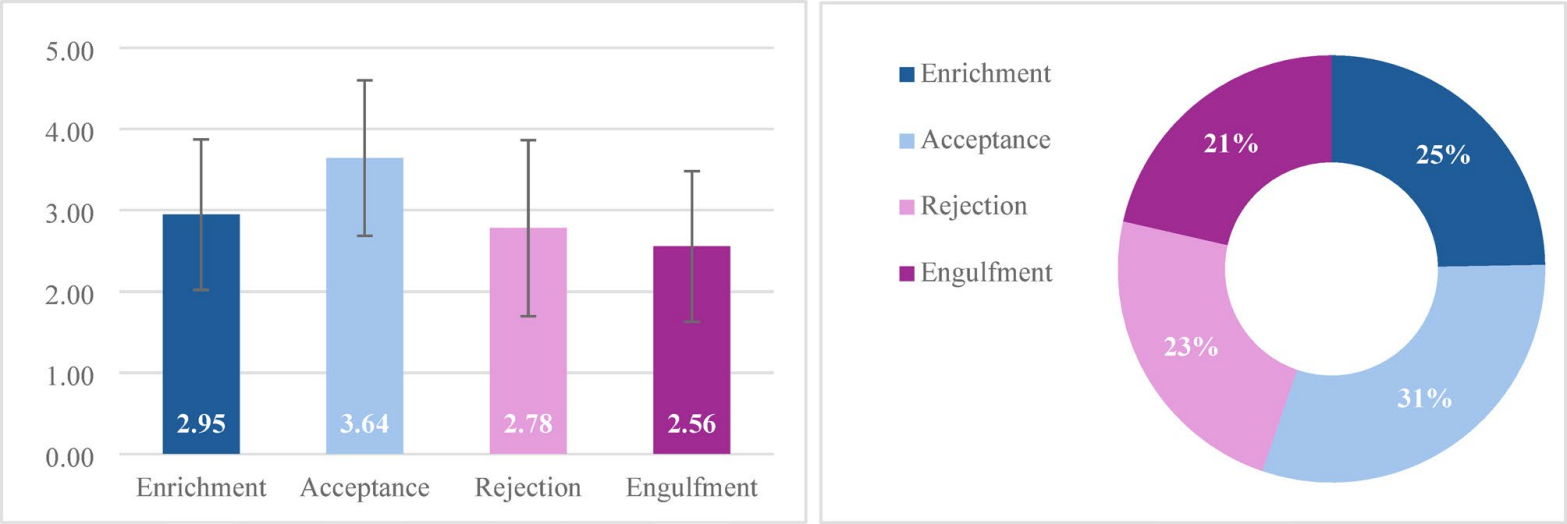
The IIQ dimension comparisons and a normalized distribution of the IIQ dimension. Note. IIQ = Illness identity questionnaire.

A repeated-measures ANOVA revealed a significant main effect for the IIQ dimensions, *F*(2.20, 459.17) = 33.20, *p* <.001, η²ₚ = 0.18, with Greenhouse-Geisser correction applied due to a violation of the sphericity assumption, as indicated by Mauchly’s test, *χ*²(5) = 76.24, *p* <.001. Post hoc pairwise comparisons using the Bonferroni correction showed that *acceptance* had significantly higher scores than all other subscales (*p* <.001 for all pairwise comparisons). *Enrichment* was rated significantly higher than *engulfment* (*p* <.01). No difference was found between *rejection* and *engulfment*. Figure 1 illustrates these findings, presenting a bar chart of the dimension comparisons and a doughnut chart showing the normalized distribution of each identity dimension relative to the total construct.

### Correlations between ADHD identity perception and symptom severity

The total ASRS score ranged from 1 to 71, with a mean score of 41.10 (*SD* = 11.56), suggesting elevated ADHD-related symptoms in the sample. The inattention subscale ranged from 1 to 35, with a mean score of 21.98 (*SD* = 6.12), indicating elevated inattentive symptoms. The hyperactivity/impulsivity subscale ranged from 0 to 36, with a mean score of 19.12 (*SD* = 6.78), suggesting notable hyperactive and impulsive behaviors.

A small but statistically significant negative correlation was found between the total ASRS score and the total IIQ score (*r* = –.18, *p* <.05), indicating that higher ADHD symptom severity was associated with a less positive ADHD identity perception. A follow-up analysis examined the associations between the ADHD symptom subscales (inattention and hyperactivity/impulsivity) and the IIQ’s four dimensions. This analysis revealed that *engulfment* was moderately positively correlated with inattention symptoms (*r* =.34, *p* <.001) and showed a weaker, yet significant, correlation with hyperactivity/impulsivity symptoms (*r* =.26, *p* =.001). This indicates that participants reporting higher levels of ADHD symptoms also tended to experience a stronger sense of being engulfed by the diagnosis. No significant associations were found between either ASRS subscale and the *rejection*, *acceptance*, or *enrichment* dimensions of the IIQ.

### Correlations between ADHD identity perception and HRQoL

To examine the relationship between ADHD identity perception and HRQoL, Pearson’s correlation was calculated. A significant positive association was found between the total IIQ score and the total PedsQL score (*r* =.40, *p* <.001), indicating that a more adaptive diagnosis identity was associated with a higher overall QoL. Detailed correlations between each IIQ dimension and PedsQL domain are presented in Table 1.

**Table 1:** Correlations between the illness identity questionnaire (IIQ) and the pediatric quality of life inventory (PedsQL).

		PedsQL			
		Physical	Emotional	Social	School
IIQ	*Rejection*	− 0.11	− 0.16	− 0.43***	− 0.11
	*Engulfment*	− 0.28**	− 0.42***	− 0.36***	− 0.36***
	*Acceptance*	0.10	0.11	0.28**	0.11
	*Enrichment*	0.15	− 0.01	0.11	0.02

Note. Bold values indicate significant effects. **p <.01; ***p <.001.

### Predicting quality of life by ADHD identity and ADHD symptom severity

A stepwise multiple regression examined which variables best predict overall QoL. In the first step, *engulfment* was entered and significantly predicted overall QoL, *F*(1, 139) = 42.42, *p* <.001, explaining 23.4% of the variance (*R*² = 0.234, adjusted *R*² = 0.228), as higher levels of *engulfment* feelings predicted lower QoL (*B* = −6.66, *SE* = 1.02, β = − 0.48). In the second step, inattentive ADHD symptoms were added to the model, resulting in significant improvement: Δ*R*² = 0.102, *F* Change(1, 138) = 21.22, *p* <.001. The final model explained 33.6% of the variance (adjusted *R*² = 0.326), *F*(2, 138) = 34.91, *p* <.001. Table 2 presents the full regression coefficients.

**Table 2:** Stepwise multiple regression predicting overall quality of Life.

Step	Predictor	B (Unstandardized)	SE B	β (Standardized)	t	*p*
1	*Engulfment*	−6.66	1.02	−0.48	−6.51	< 0.001
	*R*² = 0.234					
	Adjusted *R*² = 0.228					
	*F*(1, 139) = 42.42, *p* <.001					
2	*Engulfment*	−5.18	1.01	−0.38	−5.14	< 0.001
	ADHD Inattentive	−0.69	0.15	−0.34	−4.61	< 0.001
	*R*² = 0.336					
	Adjusted *R*² = 0.326					
	*F*(2, 138) = 34.91, *p* <.001					

Note. ADHD = attention deficit hyperactivity disorder.

### Predicting ADHD identity by ADHD symptom severity and quality of life

A stepwise multiple regression analysis determined which ADHD symptom severity (inattentive and hyperactive/impulsive subscales) and PedsQL domain (physical, emotional, social, and school) best explained the variance in adolescents’ diagnostic-identity perception (IIQ total score). Only the social PedsQL domain emerged as a significant predictor of diagnostic identity, *F*(1, 139) = 34.13, *p* <.001, and accounted for 19.7% of the variance in the IIQ (*R*² = 0.197, adjusted *R*² = 0.191). A higher social QoL was predicted to be associated with a more adaptive diagnostic identity (*B* = 0.015, *SE* = 0.003, β = 0.44; Table 3).

**Table 3:** Stepwise multiple regression predicting diagnostic identity (IIQ total Score).

Step	Predictor	B (Unstandardized)	SE B	β (Standardized)	t	*p*
1	Social QoL	0.015	0.003	0.003	5.842	< 0.001
	*R*² = 0.197					
	Adjusted *R*² = 0.191					
	*F*(1, 139) = 34.13, *p* <.001					

Note. QoL = quality of life.

### ADHD identity dimensions as mediators between ADHD symptom severity and quality of life

**Fig. 2:**
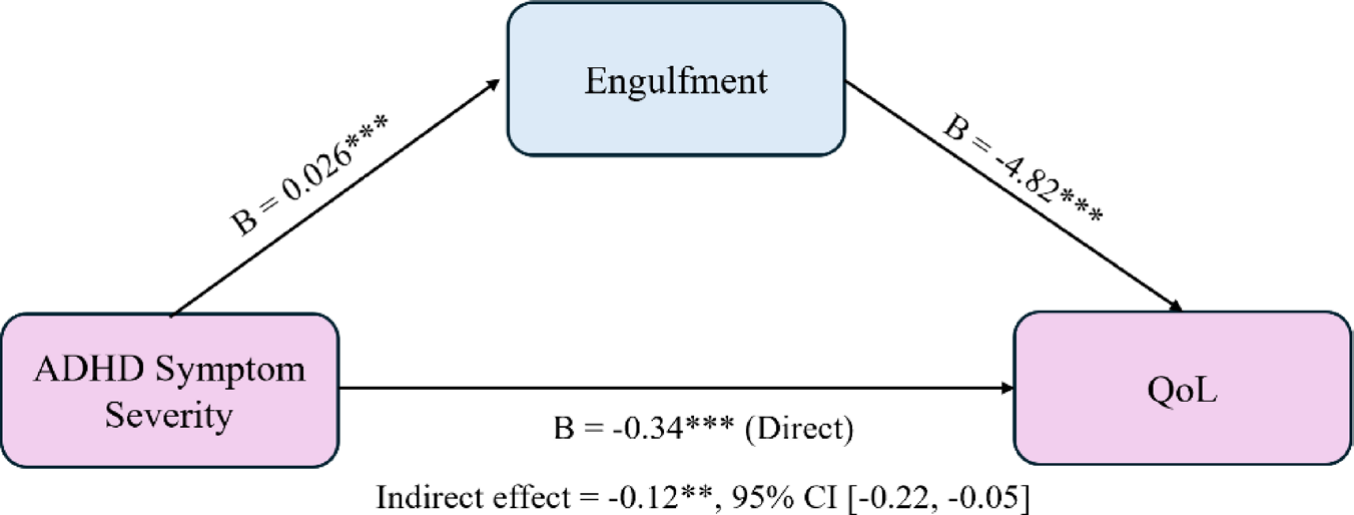
Mediation model: engulfment mediates the effect of ADHD symptom severity on QoL.

To examine whether the IIQ dimensions mediate the relationship between ADHD symptom severity and HRQoL, a parallel mediation analysis was conducted. The total effect of ADHD symptom severity on HRQoL was significant, *c* = −0.47, *p* <.001. After accounting for the four IIQ dimensions as mediators, the direct effect remained significant, $c^\prime$ = − 0.34, *SE* = 0.08, *t* = −4.19, *p* <.001, indicating partial mediation. Among the four proposed mediators, only *engulfment* demonstrated a significant indirect effect, *B* = −0.12, Boot*SE* = 0.04, 95% CI [−0.22, −0.05], suggesting that greater ADHD symptom severity predicts higher *engulfment* feelings, which are associated with lower QoL. The indirect effects through *rejection*, *acceptance*, and *enrichment* were not significant (see Table 4; Fig. 2).

**Table 4:** Indirect effects of ADHD symptoms on quality of life via diagnosis identity dimensions.

Effect type	Mediator	Effect (B)	BootSE	95% CI Lower	95% CI Upper	*p* value
Indirect effect	*Rejection*	−0.0098	0.0165	−0.0494	0.0175	> 0.05
Indirect effect	*Acceptance*	−0.0024	0.0108	−0.0249	0.0206	> 0.05
Indirect effect	*Engulfment*	−0.1232	0.0429	−0.2183	−0.0512	**< 0.01**
Indirect effect	*Enrichment*	0.0002	0.0073	−0.0170	0.0150	> 0.05
Total indirect	—	−0.1352	0.0504	−0.2448	−0.0448	**< 0.01**
Direct effect	—	−0.3353	0.0801	−0.4937	−0.1769	**< 0.001**
Total effect	—	−0.4705	—	—	—	**< 0.001**

Note. ADHD = attention deficit hyperactivity disorder. Bold values indicate significant effects.

## Discussion

The present study aimed to examine how adolescents with ADHD perceive their diagnostic identity and how these perceptions are associated with ADHD symptom severity and HRQoL. Although prior qualitative studies have highlighted the potential impact of an ADHD diagnosis on adolescents’ identity formation, the present cross-sectional study offers an initial quantitative exploration of this phenomenon using the IIQ adapted for ADHD. By exploring the role of diagnostic identity concerning symptom severity and well-being, the current findings contribute to a broader understanding of the psychosocial processes influencing adolescents with ADHD. These insights may inform future clinical approaches, supporting more individualized interventions that consider symptom management and the adolescent’s self-concept and QoL.

### Diagnostic identity profile in adolescents with ADHD

The findings suggest a general pattern of diagnostic identity among adolescents with ADHD, with higher mean levels of *acceptance* and *enrichment* and lower levels of *engulfment* and *rejection*. The comparative analysis of identity dimensions highlights which identity dimensions are comparatively more or less salient for adolescents with ADHD (e.g., higher acceptance vs. lower engulfment), providing clinically meaningful insight into areas that may warrant greater support. This pattern aligns with prior qualitative research, which suggested that many adolescents perceive ADHD as an important part of their self-definition, but not one that wholly dominates their identity^24,25^. Similarly, recent studies have indicated that neurodiverse youth are increasingly likely to articulate a sense of agency and personal meaning regarding their diagnoses, reflecting a shift toward more empowered narratives^51,52^.

The relatively high endorsement of *enrichment* may also reflect broader sociocultural changes, including the rise of neurodiversity-affirming perspectives in educational and social discourse^52,53^. Exposure to inclusive environments may enable adolescents to reframe their ADHD not as a deficit but as a potential source of personal growth, creativity, or unique social contributions.

Importantly, some adolescents reported high *acceptance* alongside persisting feelings of *engulfment*, indicating that elements of identity integration may co-exist rather than being mutually exclusive. This suggests that for some adolescents, integrating the ADHD diagnosis into their identity involves a dynamic and ongoing negotiation, rather than a static or dichotomous process. These findings are consistent with contemporary models of identity integration in youth with chronic conditions, which conceptualize identity formation as multidimensional and fluid^15,31^. Thus, the present study underscores the importance of assessing diagnostic identity as a spectrum, recognizing that individuals within the same diagnostic category may experience their condition in diverse and sometimes ambivalent ways.

### ADHD symptom severity and diagnostic identity

The observed association between greater ADHD symptom severity and higher levels of *engulfment*, but not with *rejection*, *acceptance*, or *enrichment*, adds granularity to existing literature. Previous studies have linked increased ADHD symptoms to lower self-esteem and higher internalized stigma^54,55^, but few have directly examined this link through the lens of diagnostic identity. Our findings suggest that symptom severity may specifically contribute to adolescents perceiving their ADHD as dominating or all-defining, a process conceptualized in the diagnosis identity model as *engulfment*. Notably, the association between *engulfment* and inattention symptoms was stronger than with hyperactivity/impulsivity. This pattern may reflect the pervasive and internalized nature of attentional difficulties, which often interfere with multiple life domains in ways that are difficult to externalize or attribute to situational factors^9,10^. In contrast, hyperactive or impulsive behaviors may be more observable and situationally triggered, making them less likely to be incorporated into the core self-concept.

These findings align with contemporary models of self-labeling and identity development, which have suggested that when adolescents repeatedly experience disruptions in daily functioning due to uncontrollable symptoms, they may begin to over-identify with diagnostic labels, increasing the risk of *engulfment*^56,57^. Clinically, this underscores the need to address symptom severity’s affective and identity-related consequences, not merely behavioral management.

Interestingly, no significant associations were observed between ADHD symptom severity and the other diagnostic identity dimensions (acceptance, rejection, and enrichment). This may suggest that these dimensions are shaped less by symptom intensity and more by contextual and interpersonal factors, such as supportive environments or experiences of stigma. Prior work has emphasized that positive dimensions of illness/diagnosis identity, such as acceptance and enrichment, are often fostered by supportive relationships and inclusive environments rather than by symptom reduction^31,63^. Similarly, rejection may be more strongly linked to experiences of stigma than to the severity of ADHD symptoms themselves^24^. These findings highlight the importance of considering social and contextual influences, in addition to clinical severity, when examining how adolescents integrate ADHD into their sense of self.

### ADHD identity and HRQoL

Diagnostic identity, particularly the *engulfment* dimension, was significantly associated with lower HRQoL across multiple domains. These findings extend previous work in youth with epilepsy and diabetes, where Engulfment similarly predicted poorer well-being^15,31^. Our study adds novel evidence that identity processes are relevant not only in chronic physical conditions but also in neurodevelopmental disorders such as ADHD.

A more detailed examination of HRQoL domains reveals important nuances. *Engulfment* was associated with a lower QoL in physical, emotional, social, and academic contexts. This likely reflects the pervasiveness of an engulfed identity, wherein the diagnosis is perceived as affecting all life domains, leading to a global reduction in well-being^9^. In contrast, *rejection* and *acceptance* were specifically linked to social QoL, suggesting that how adolescents manage or disclose their diagnosis may primarily impact their social interactions and sense of belonging^24^.

Interestingly, *acceptance* alone was not strongly associated with overall HRQoL, challenging prior assumptions that it is inherently protective of overall HRQoL. This may indicate that *acceptance* without positive meaning-making (*enrichment*) and without minimizing *engulfment* may not be sufficient to enhance well-being. Furthermore, adolescents may interpret *acceptance* in varied ways—active integration or passive resignation—a distinction increasingly recognized in illness identity research^58^. According to cognitive appraisal models, it is not the diagnosis itself but the individual’s interpretation of it that shapes well-being^59^.

### The role of engulfment and inattention in predicting HRQoL

Regression analyses revealed that *engulfment* accounted for more variance in HRQoL than ADHD symptom severity, underscoring the importance of identity processes in shaping adolescents’ well-being. These findings align with contemporary models in health psychology, where identity centrality, not symptom severity per se, often drives psychosocial outcomes^57,60,61^. The current results extend this model to ADHD, suggesting that when adolescents perceive their diagnosis as all-defining, their well-being deteriorates across emotional, social, and functional domains.

Importantly, inattention symptoms also emerged as a significant predictor of reduced HRQoL. This finding is consistent with previous research, which indicates that inattentive symptoms disrupt daily functioning, affecting school performance, social interactions, and emotional regulation^10^. Unlike hyperactivity, inattention often leads to internalized self-perceptions of inadequacy, which may compound the negative effects on QoL^9^.

Clinically, these findings underscore the need to address maladaptive identity integration, rather than merely controlling symptoms. Traditional interventions focusing solely on behavior management may fall short if adolescents continue to perceive their ADHD as a central and limiting aspect of their identity. As highlighted in recent conceptual reviews, identity-targeted approaches, such as narrative therapy, peer mentoring, and identity-mapping tools, can enhance outcomes in this population^58,62^.

### Social quality of life as a predictor of ADHD identity

This study’s novel contribution is identifying social QoL as a significant predictor of diagnostic identity, independent of symptom severity. This finding highlights the role of interpersonal experiences, such as peer acceptance, social exclusion, or teacher relationships, in shaping how adolescents internalize their diagnosis. These results align with those of Hutchinson and Banerjee^63^, who found that adolescents with neurodevelopmental conditions develop more integrated identities when they experience social inclusion. In contrast, social exclusion can reinforce stigmatized self-perceptions and exacerbate *rejection* or *engulfment*. Their findings suggested that social participation is not merely an outcome of adaptive identity but also a driver of it, aligning with theories of self-verification and identity negotiation^64,65^. Clinically, this emphasizes fostering social inclusion through school and community-based supports to promote healthier identity development in adolescents with ADHD.

### Engulfment as a mediator between ADHD symptoms severity and quality of life

The mediation analysis revealed that *engulfment* partially explained the relationship between ADHD symptom severity and lower HRQoL. This reinforces the role of identity as a psychological pathway through which objective impairments translate into subjective distress. These findings align with recent theoretical advances proposing that identity functions as a “gateway variable” in health psychology, linking biological, psychological, and social processes^66^. This pattern is consistent with models of illness centrality and engulfment, suggesting that when a diagnosis becomes overly central to one’s self-concept, global reductions in well-being occur regardless of symptom severity^60,61^. The lack of mediation by *acceptance*, *rejection*, or *enrichment* suggests that *engulfment* uniquely captures the maladaptive internalization of ADHD symptoms into identity, resulting in diminished QoL.

This is the first study to empirically demonstrate this mediation mechanism in adolescents with ADHD, highlighting the diagnostic identity construct as both theoretically robust and clinically relevant. These findings support previous research in chronic conditions showing that identity-related engulfment is a robust predictor of psychological distress and reduced functioning^57,58^. These findings can also be interpreted within broader theoretical frameworks of mental health and developmental psychopathology. According to Keyes’ Mental Health Continuum model^67^, well-being reflects both the absence of risk factors (e.g., negative identity perceptions) and the presence of adaptive qualities (e.g., positive identity perceptions). Similarly, developmental models of ADHD^68^ conceptualize adjustment as a balance between risk and resilience processes across adolescence. The current results align with these frameworks, suggesting that identity integration may represent a key mechanism through which adolescents move along the continuum from languishing to flourishing. Clinically, this underscores the need for identity-focused interventions. Techniques such as narrative therapy, identity mapping, and cognitive reframing of diagnostic labels may help reduce *engulfment*, thereby buffering the psychological impact of a high symptom burden^62^. Future research should explore the longitudinal trajectories of identity formation and test whether targeted reductions in *engulfment* can improve outcomes for adolescents living with ADHD.

### Conclusions and clinical implications

This study highlights the crucial role of diagnostic identity, particularly the dimension of Engulfment, in influencing adolescents’ HRQoL beyond the direct effects of symptom severity. These findings suggest that effective interventions for adolescents with ADHD must extend beyond symptom management and explicitly address how young people perceive, narrate, and integrate their diagnosis into their self-concept. Importantly, adolescents often experience ADHD as a dual narrative, simultaneously perceiving it as a source of vulnerability and unique personal strengths such as creativity, high energy, and humor^24,27^. Clinical care should support adolescents in balancing these perspectives, promoting identity development that acknowledges challenges while fostering resilience and self-acceptance. Furthermore, the results underscore the significance of social experiences in shaping identity formation. Interventions that enhance social inclusion—school-based programming, peer mentoring, or community-level initiatives—may indirectly support healthier identity integration and improved well-being^63^. Clinicians should also consider how diagnostic identity perceptions may influence treatment decisions, including medication adherence. Prior qualitative research suggests that some adolescents discontinue pharmacological treatment to preserve a sense of autonomy or protect their “authentic self” from being medicated^23,26^. Thus, psychoeducation and therapeutic dialogues should incorporate identity-focused discussions, enabling adolescents and their families to make treatment decisions that align with personal values while maintaining a focus on holistic health. Integrating these considerations into clinical practice may help guide more person-centered, developmentally attuned approaches for adolescents with ADHD, although causal relationships cannot be inferred due to the study’s cross-sectional design.

### Limitations and future directions

Several limitations of this study should be acknowledged. The cross-sectional design precludes the conclusion of causality or developmental trajectories. Additionally, although no missing data occurred due to the mandatory response setting in the online survey, this approach may have introduced a potential selection bias. Participants who were unwilling or unable to complete all items were automatically excluded, possibly leading to a sample that is more conscientious, motivated, or engaged than the broader population of individuals with ADHD. While the findings suggest associations between ADHD symptom severity, diagnostic identity, and QoL, longitudinal studies are necessary to determine how these relationships evolve, particularly during key periods of identity formation in adolescence and early adulthood.

The data relied exclusively on self-report measures, which may limit verification of participant identity and introduce biases such as social desirability or subjective misperceptions of symptoms and identity. Incorporating multi-informant reports (e.g., parents, teachers, clinicians) and qualitative methodologies could enrich the understanding of adolescents’ lived experiences and reduce measurement bias. The study sample consisted solely of adolescents with ADHD diagnoses without comorbidities, which, although necessary for isolating ADHD-specific effects, limits the generalizability of findings to the broader ADHD population in which comorbidity is the norm. Future research should explore how comorbid conditions such as anxiety, depression, or autism spectrum disorder may interact with diagnostic identity formation and well-being. Additionally, cultural and contextual factors may shape how adolescents perceive and integrate their ADHD diagnosis into their identity. Because the sample was recruited from a single cultural context, cross-cultural research could provide insights into the role of social norms, stigma, and neurodiversity narratives in shaping diagnostic identity.

The use of stepwise regression carries the risk of overfitting and sample-specific results. Therefore, the predictors identified in this study should be interpreted with caution and replicated in future studies using larger samples and alternative analytic approaches. Finally, although this study focused on the *engulfment* dimension as a mediator, further research is warranted to explore whether interventions aimed at reducing *engulfment* and fostering *enrichment* or *acceptance* can improve long-term outcomes. Experimental and interventional studies, such as trials of identity-focused therapies or peer support groups, could clarify whether modifying diagnostic identity mitigates the psychosocial impact of ADHD.

## Data Availability

The datasets generated during and/or analysed during the current study are not publicly available due to ethical limitations but are available from the corresponding author on reasonable request.

## Abbreviations

ADHD: attention deficit hyperactivity disorder
ANOVA: analysis of variance
ASRS: Adult ADHD Self-Report Scale
HRQoL: health-related quality of life
IIQ: Illness Identity Questionnaire
PedsQL: Pediatric Quality of Life Inventory
QoL: quality of life
